# Evaluation of Oxygen Saturation Index Compared With Oxygenation Index in Neonates With Hypoxemic Respiratory Failure

**DOI:** 10.1001/jamanetworkopen.2019.1179

**Published:** 2019-03-29

**Authors:** Hemananda K. Muniraman, Ashley Y. Song, Rangasamy Ramanathan, Kathryn L. Fletcher, Rutuja Kibe, Li Ding, Ashwini Lakshmanan, Manoj Biniwale

**Affiliations:** 1 Neonatology Association Limited, Obstetrix Medical Group of Phoenix, Mednax, Phoenix, Arizona; 2 Division of Neonatal-Perinatal Medicine, Department of Pediatrics, Los Angeles County+USC Medical Center, Keck School of Medicine, University of Southern California, Los Angeles; 3 Department of Preventive Medicine, Keck School of Medicine, University of Southern California, Los Angeles; 4 Fetal and Neonatal Institute, Division of Neonatology, Children’s Hospital Los Angeles, Department of Pediatrics, Keck School of Medicine, University of Southern California, Los Angeles; 5 Schaeffer Center for Health Policy and Economics, University of Southern California, Los Angeles

## Abstract

**Importance:**

Oxygenation index (OI), an invasive measurement, is routinely used as a marker of severity of hypoxemic respiratory failure in neonates. Oxygen saturation index (OSI) is a noninvasive measurement and has been shown to be a reliable surrogate marker of OI in children and adults with respiratory failure.

**Objectives:**

To evaluate the correlation of OI with OSI and to derive and validate predictive OI from noninvasive OSI measurements for clinically relevant OI values.

**Design, Setting, and Participants:**

For this retrospective cohort study, 220 neonates requiring invasive mechanical ventilation for hypoxic respiratory failure during the first 3 days of admission were recruited from a level III neonatal intensive care unit during a 6-year period, from January 1, 2012, to December 31, 2017. Data were analyzed from January 2017 to December 2017.

**Main Outcomes and Measures:**

The primary outcome was correlation of OI with OSI, analyzed using Pearson correlation coefficient. The secondary outcome was derivation and validation of OI from OSI. The data were split into derivative samples, from which a predictive equation for OI was derived using generalized linear model, and a validation sample was used to assess the predictive ability of the derived OI. Bland-Altman plot was used to assess agreement between derived OI and measured OI.

**Results:**

A total of 1442 paired OI and OSI measurements from 220 infants (190 preterm and 30 term; median [interquartile range] gestational age, 29 [26-33] weeks; mean [SD] birth weight, 1602 [1092] g) were recorded during the study. The median (interquartile range) number of samples was 5 (3-9) per patient. Overall, OI and OSI showed strong correlation (*r* = 0.89). The correlation was stronger in preterm infants (<28 weeks, *r* = 0.93; 28-33 weeks, *r* = 0.93) and within an oxygen saturation range of 85% to 95% (*r* = 0.94). The predictive derivative equation showed a strong linear association and good agreement in both derivation and validation data sets, with strong accuracy measures of derived OI for OI cutoffs of 5, 10, 15, 20, and 25.

**Conclusions and Relevance:**

A strong correlation of OI with OSI was found. Derived OI from OSI was in good agreement and strongly predictive of clinically relevant OI cutoffs from 5 to 25. Oxygenation index derived from noninvasive sources may be useful to reliably assess severity of respiratory failure and response to therapy on a continuous basis.

## Key Points

**Question:**

Is oxygen saturation index (OSI) a reliable surrogate marker of oxygenation index (OI) in neonates with hypoxemic respiratory failure?

**Findings:**

In this cohort study including 1442 paired OI and OSI measurements from 220 neonates, OSI was noted to correlate strongly with OI. Derived OI from OSI was in good agreement and strongly predictive of clinically relevant OI cutoffs from 5 to 25.

**Meaning:**

Derived OI from noninvasive measurements may be useful to reliably assess severity of respiratory illness and response to therapy on a continuous basis.

## Teaser

This cohort study assesses the agreement of oxygenation index with measurements derived from oxygen saturation index in neonates with hypoxic respiratory failure.

## Introduction

Hypoxemic respiratory failure (HRF) is one of the common reasons in neonates for admission to neonatal intensive care units in the United States. Estimated incidence of neonates with respiratory failure requiring mechanical ventilation is approximately 18 per 1000 live births.^1,2^ Hypoxemic respiratory failure is associated with increased risk of mortality, morbidity, and worse neurological outcomes.^3,4^ Oxygenation index (OI) is routinely used as an indicator of severity of HRF in neonates, with an arbitrary cutoff of 15 or less for mild HRF, between 16 and 25 for moderate HRF, between 26 and 40 for severe HRF, and more than 40 for very severe HRF.^5^ Oxygenation index is calculated as OI = MAP × Fio_2_ × 100 / Pao_2_, where MAP indicates mean airway pressure and Fio_2_ indicates fraction of inspired oxygen.^5^ Oxygenation index has been used as a marker in clinical management and clinical trials for initiating therapies including inhaled nitric oxide in infants with HRF and pulmonary hypertension^6,7,8,9^ and for administering and evaluating response to surfactant therapy.^10,11^ Oxygenation index higher than 40 is used as a criterion for consideration of extracorporeal membrane oxygenation.^12,13^ Oxygenation index has also been proposed as a predictive marker for neonatal outcomes, including mortality.^14^ Limitations of OI include the need for an indwelling arterial catheter for frequent sampling and that it is an intermittent measurement of oxygenation status by nature. Oxygen saturation index (OSI) replaces Pao_2_ with oxygen saturation as measured by pulse oximetry (Spo_2_) in the OI equation and is calculated as OSI = MAP × Fio_2_ × 100 / Spo_2_.^15,16^ The advantages of OSI include that it is noninvasive and allows continuous monitoring of oxygenation status. Oxygen saturation index has been validated in pediatric intensive care unit patients as a reliable index for assessing severity of respiratory failure and lung injury.^15,16^ However, to our knowledge, studies on the use of OSI in neonates with HRF are very limited.^17,18^

The primary objective of this study was to evaluate the correlation of OSI with OI in preterm and term infants. Our secondary objectives were to derive OI from noninvasive OSI for clinically relevant OI cutoffs (5, 10, 15, 20, 25, and 40) and to validate the accuracy of the derived OI cutoffs using OI cutoffs as the criterion standard.

## Methods

We performed a retrospective cohort study including all neonates admitted to a single level III neonatal intensive care unit during a 6-year period (January 1, 2012, to December 31, 2017) with respiratory distress requiring invasive mechanical ventilation with indwelling arterial catheters and continuous pulse oximeter monitoring in the first 3 days of admission. We excluded infants with cyanotic congenital heart disease. Data were collected from electronic medical records and included birth weight, gestational age, time and results of arterial blood gases, source of arterial blood sampling, corresponding oxygen saturation at the time of arterial blood sampling, site of pulse oximeter, and patient temperature. It has been standard practice for respiratory therapists to record location and the precise Spo_2_ measurement in electronic medical records at the time of blood gas sampling. Paired OI and OSI data were calculated from Pao_2_ and Spo_2_, respectively, at the time of arterial blood sampling. This study is reported according to the Strengthening the Reporting of Observational Studies in Epidemiology (STROBE) reporting guideline. The University of Southern California Health Sciences institutional review board approved this study with waiver of informed consent.

Data were analyzed from January 2017 through December 2017. Correlation of OI with OSI was analyzed using Pearson correlation. For the correlation, we log-transformed data for OI and OSI. Correlation was analyzed for the whole cohort of paired samples and stratified based on source of arterial blood sampling and pulse oximeter site (preductal or postductal), gestational age groups (extremely preterm [<28 weeks], moderately preterm [28-33 weeks], late preterm [34-36 weeks], and term gestation [≥37 weeks]),^19^ and different Spo_2_ ranges.

A predictive equation for the association of OSI with OI was derived by linear regression. Since data included repeated measurements of arterial blood sampling and Spo_2_ over different times in each patient, generalized linear models (generalized estimating equations) were used to account for repeated measurements within individual patients.^20^ The whole data set was split using computer-generated random assignment into a derivation data set for prediction model building (70%) and a validation data set (30%) for validation of the derived regression equation.^16^

Derived OI was calculated from the regression equation for clinically relevant OI cutoffs (5, 10, 15, 20, 25, and 40). Multivariate mixed-modeling analysis was performed to control for plausible variables that are known to affect the relationship between Pao_2_ and Spo_2_ in the oxygen dissociation curve, namely pH, Paco_2_, and patient temperature.^21^ The discriminative ability of derived OI for clinically relevant OI cutoffs was analyzed using accuracy measures, including sensitivity, specificity, positive predictive value, negative predictive value, and area under the curve, for the derivation data and validated using the validation data.

 Agreement of derived OI from the regression equation using OSI with measured OI calculated from arterial blood sampling was evaluated by Bland-Altman method from the derived data and validated with the validation data. Agreement of the 2 measurements was also assessed within different Spo_2_ and OI ranges.

Correlation was considered significant at *P* < .01 (2-tailed test of probability). SPSS Statistics for Windows version 25.0 (IBM Corp), SAS software version 9.4 (SAS Institute), and MedCalc for Windows version 15.0 (MedCalc Software) were used for analyses.

## Results

Thirty infants admitted during the study period were excluded because they did not have access to indwelling arterial catheters. Twelve paired samples were excluded from the correlation analysis for incomplete patient characteristic data. A total of 1442 paired samples from 220 neonates (190 preterm and 30 term) were recorded during the 6-year study period. The median (interquartile range) number of samples was 5 (3-9) per patient. The median (interquartile range) gestational age was 29 (26-33) weeks, with a mean (SD) birth weight of 1602 (1092) g. 

Oxygen saturation index correlated strongly with OI (*r* = 0.89) for the whole cohort (Table 1). Oxygen saturation index calculated from both preductal and postductal sources of Spo_2_ showed strong correlation with OI calculated from Pao_2_ obtained from an umbilical arterial source (Table 1). Oxygenation index calculated from peripheral arterial line sources correlated strongly with OSI derived from both preductal Spo_2_ (n = 45; *r* = 0.86) and postductal Spo_2_ (n = 154; *r* = 0.94) sources. Oxygen saturation index strongly correlated with OI within the Spo_2_ range of 85% to 95% (*r* = 0.94). Correlation for ranges more than 95% or less than 65% (n = 9, *r* = 0.75) was poor (Table 1). Oxygen saturation index correlated more strongly in preterm infants with gestational ages less than 34 weeks (<28 weeks, *r* = 0.93; 28-33 weeks, *r* = 0.93) compared with late preterm (*r* = 0.86) and term (*r* = 0.70) infants (Table 1).

**Table 1.** Correlation of Oxygenation Index With Oxygen Saturation Index Based on Oxygen Saturation

Measure	Measurements, No.	Pearson *r* Correlation	*P* Value^a^
Total sample	1442	0.89	<.001
UA Pao_2_ compared with Spo_2_^b^			
From preductal source	142	0.94	<.001
From postductal source	435	0.94	<.001
Spo_2_, %			
<85	146	0.90	<.001
85-95	635	0.94	<.001
>95	661	0.70	<.001
Infant GA, wk			
<28	745	0.93	<.001
28-33	377	0.93	<.001
34-36	136	0.86	<.001
≥37	184	0.70	<.001

Abbreviations: UA, umbilical artery; GA, gestational age; Spo_2, _oxygen saturation as measured by pulse oximetry.

^a^ Significant at *P* = .01 (2-tailed test of probability).

^b^ Infants lacking information on UA Pao_2_ or source of Spo_2 _were excluded, leaving 577 matched pairs for analysis.

The regression equation from the derivation data set (OI = 0.0745 + 1.7830 × OSI) showed strong linear association of OSI with OI . Multivariate linear modeling showed OI was associated with OSI, but pH, Paco_2_, and temperature were not significantly associated with OSI and OI (eTable 1 in the Supplement). Baseline characteristics were similar in the derivation and validation data sets, with the exception of higher hemoglobin levels in the derivation data subset (14.23 g/dL vs 13.80 g/dL [to convert to grams per liter, multiply by 10]) (eTable 2 in the Supplement).

The accuracy measures from the derivation data evaluating the ability of the derived OI to discriminate clinically significant OI cutoffs and ruling in patients above the corresponding OI cutoff were good, with an area under the curve greater than 0.85 for OI cutoffs 5, 10, 15, 20, and 25 and high negative predictive values and specificities. However, for OI cutoffs more than 40, the discriminating ability was poor, with an area under the curve less than 0.7, although the specificity and negative predictive value were more than 98% (Table 2). The accuracy measures were similar when evaluated for the validation sample (Table 2).

**Table 2.** Accuracy Measures Based on Derived Values of OI^a^

Cutoff	AUC	%			
		Sensitivity	Specificity	PPV	NPV
Derived OI ≥5					
Derivation (n = 451)	0.88	85	91	88	89
Validation (n = 200)	0.88	87	90	88	89
Derived OI ≥10					
Derivation (n = 173)	0.84	74	96	80	95
Validation (n = 76)	0.85	74	95	77	94
Derived OI ≥15					
Derivation (n = 101)	0.91	77	98	79	97
Validation (n = 46)	0.86	74	97	76	97
Derived OI ≥20					
Derivation (n = 74)	0.91	84	98	78	99
Validation (n = 32)	0.87	75	98	78	98
Derived OI ≥25					
Derivation (n = 53)	0.85	72	98	71	98
Validation (n = 17)	0.88	76	98	65	99
Derived OI ≥40					
Derivation (n = 22)	0.61	23	99	39	98
Validation (n = 9)	0.67	33	99	50	99

Abbreviations: AUC, area under receiver operative curve; NPV, negative predictive value; OI, oxygenation index; PPV, positive predictive value.

^a^ Sensitivity, specificity, PPV, and NPV using the derived OI equivalents for clinically relevant OI cutoffs from the derived data set applied to both derived and validation data sets.

**Figure 1.**
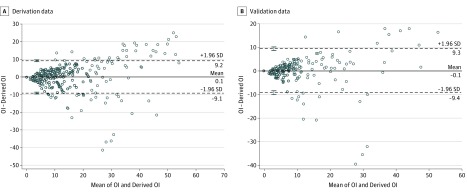
Bland-Altman Analysis Comparing Derived Oxygenation Index (OI) With Measured OI From Pao_2_ Dashed lines indicate limits of agreement; error bars, SD of limits of agreement.

**Figure 2.**
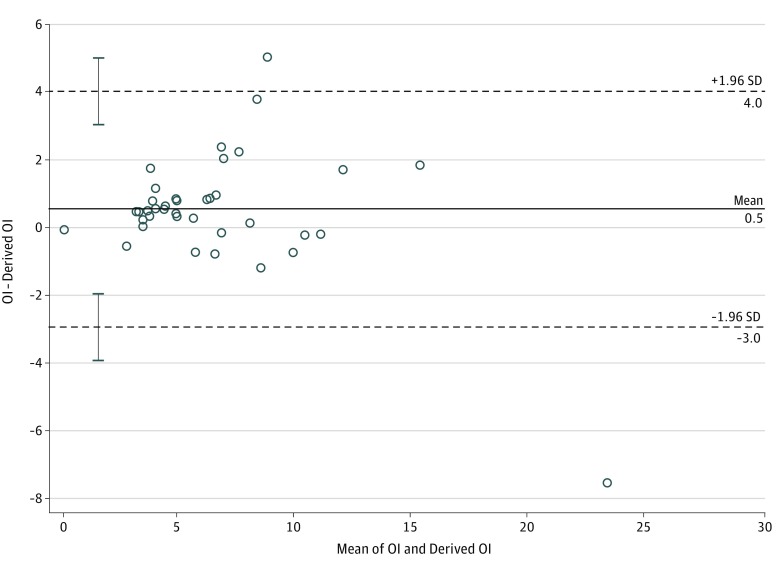
Bland-Altman Plot Comparing Derived Oxygenation Index (OI) and Measured OI in Term Infants Scatterplot compares derived OI and measured OI in infants with gestational age 37 weeks or older, oxygen saturation as measured by pulse oximetry range from 85% to 95%, and OI below 25. Dashed lines indicate limits of agreement; error bars, SD of limits of agreement.

**Figure 3.**
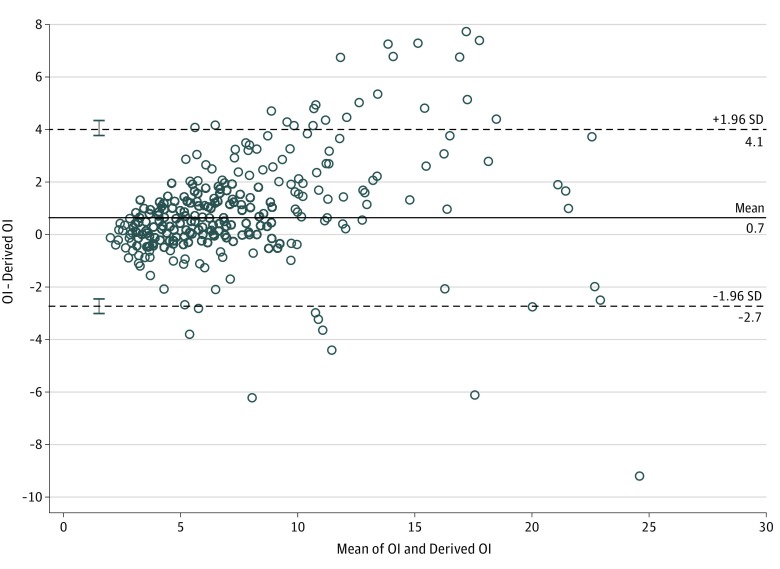
Bland-Altman Plot Comparing Derived Oxygenation Index (OI) and Measured OI in Preterm Infants Scatterplot compares derived OI and measured OI in neonates with gestational age of less than 36 weeks, oxygen saturation as measured by pulse oximetry range from 85% to 95%, and OI below 25. Dashed lines indicate limits of agreement; error bars, SD of limits of agreement.

Bland-Altman analysis of the derivative data comparing derived OI and measured OI from Pao_2_ demonstrated no significant systematic bias, with a mean of 0.1 and limits of agreement between −9.1 and 9.2 (Figure 1A). Bland-Altman analysis of the validation data presented a near-identical plot (Figure 1B). Figure 2 and Figure 3 present Bland-Altman plots for term and preterm infants, respectively, with the Spo_2_ range of 85% to 95% and OI below 25. The scatterplots demonstrated minimal bias, but there was stronger agreement within narrower limits of agreement.

## Discussion

Our study demonstrates a strong linear association of OSI, a noninvasive measurement, with OI. We present a regression equation for deriving OI from OSI and report strong discriminative ability of our derived OI to rule in patients within the OI cutoffs of 5 to 25, with good agreement within the Spo_2_ range of 85% to 95%.

Use of OSI has been increasing in pediatric and adult intensive care units as a marker for respiratory failure as well as lung injury.^14,15^ We believe our results have clinical relevance, particularly in neonates with difficult arterial access and inability to measure Pao_2_ required for OI calculation. Oxygen saturation index can be calculated readily and continuously at the bedside, without the need for invasive blood sampling, and may be useful in identifying infants with mild to moderate HRF and evaluating response to some interventions, such as inhaled nitric oxide. Furthermore, derived OI from OSI may be incorporated into inclusion criteria for clinical trials, when absence of arterial access may otherwise preclude some infants from enrollment.

To our knowledge, this is the largest study evaluating the correlation of OSI with OI in neonates. Rawat et al,^17^ in a retrospective study of 74 late preterm and term neonates, reported strong correlation of OSI with OI (between OI of 4 and 32) and proposed a practical equation for predicting OI from OSI (OI = 2 × OSI) from a regression equation similar to that reported in our study. Doreswamy et al^18^ performed a prospective study of 54 neonates and derived predictive OSI values of 3 and 6.5 for OI values of 5 and 15, respectively, with high sensitivity and specificity. We included preterm infants in our study to evaluate the association of OSI with OI in neonates across wider ranges of gestational age and clinically relevant OI cutoffs and to enhance generalizability of our results. We evaluated the association of OI with OSI based on source of Pao_2_ and Spo_2_ to account for preductal and postductal differential saturations. Our results show good correlation of preductal and postductal Spo_2_ sources with Pao_2_ obtained from the umbilical artery, which is a common source of arterial blood sampling in neonates with respiratory failure. As the association of Spo_2_ with Pao_2_ became nonlinear in the extreme ranges of Spo_2_, our results showed weaker association and agreement of OSI with OI. The limits of agreement from our Bland-Altman analysis of the whole cohort were too wide to be clinically relevant. Hence, we performed further Bland-Altman analyses to identify conditions where the measurements showed good agreement and could be clinically applicable. We found good agreement for both preterm and term infants within the Spo_2_ range of 85% to 95% and moderate HRF (OI <25) and believe that, in the absence of invasive arterial blood gas measurements, our derived OI could be most applicable in infants with moderate HRF (OI <25) and the Spo_2_ range of 85% to 95%.

### Limitations

Our study has some limitations. Arterial blood gases were measured at the clinician’s discretion. The data on time of arterial blood gas measurement and Spo_2_ recording were collected as close to the period as possible, as documented by the respiratory therapists, and the mean time difference between measurements was estimated to be less than 1 minute based on review of electronic medical records. For pragmatic reasons, we only included infants within the first 3 days of life because this period was thought to be when infants were most likely to develop HRF with respiratory distress syndrome and/or persistent pulmonary hypertension and require frequent arterial blood sampling. We attempted to control for variables that might affect the association of OSI with OI, including pH, temperature, and Paco_2_, and result in a shift of the oxygen dissociation curve. Our results showed no associations of Pco_2,_ pH, and temperature with OI and OSI, likely because most of our study participants were preterm infants with relatively stable Pco_2,_ pH, and temperature. However, other factors, such as mode of ventilation, 2,3-diphosphoglycerate levels, the effect of blood transfusions, hypothermia, and use of inhaled nitric oxide, were not studied and may have had an effect on our results. Furthermore, most of the infants in the study were preterm and may not have had significant HRF and pulmonary hypertension compared with the term infants. This may account for our findings of stronger correlation of OI with OSI in preterm infants.

## Conclusions

This study showed a strong correlation of OI with OSI. Derived OI from OSI was in good agreement and strongly predictive of clinically relevant OI cutoffs of 5 to 25. Oxygenation index derived from a noninvasive source, such as OSI, may be useful to reliably assess severity of respiratory failure and response to therapy on a continuous basis. Further studies are needed to validate and correlate OSI with clinical illness severity and neonatal outcomes.

## Notes

Supplement. **eTable 1.** Association of OI With OSI Using Multivariate Model **eTable 2.** Baseline Characteristics for Derivation and Validation Data Sets

## References

[1] Angus DC, Linde-Zwirble WT, Clermont G, Griffin MF, Clark RH Epidemiology of neonatal respiratory failure in the United States: projections from California and New York. Am J Respir Crit Care Med. 2001;164(7):-. doi:10.1164/ajrccm.164.7.2012126 11673202

[2] Lakshminrusimha S, Saugstad OD The fetal circulation, pathophysiology of hypoxemic respiratory failure and pulmonary hypertension in neonates, and the role of oxygen therapy. J Perinatol. 2016;36(suppl 2):s3-s11. doi:10.1038/jp.2016.43 27225963

[3] Eriksen V, Nielsen LH, Klokker M, Greisen G Follow-up of 5- to 11-year-old children treated for persistent pulmonary hypertension of the newborn. Acta Paediatr. 2009;98(2):304-309. doi:10.1111/j.1651-2227.2008.01065 18976361

[4] Walsh-Sukys MC, Bauer RE, Cornell DJ, Friedman HG, Stork EK, Hack M Severe respiratory failure in neonates: mortality and morbidity rates and neurodevelopmental outcomes. J Pediatr. 1994;125(1):104-110. doi:10.1016/S0022-3476(94)70134-2 7517446

[5] Golombek SG, Young JN Efficacy of inhaled nitric oxide for hypoxic respiratory failure in term and late preterm infants by baseline severity of illness: a pooled analysis of three clinical trials. Clin Ther. 2010;32(5):939-948. doi:10.1016/j.clinthera.2010.04.023 20685502

[6] Barrington KJ, Finer N, Pennaforte T, Altit G Nitric oxide for respiratory failure in infants born at or near term. Cochrane Database Syst Rev. 2017;1:CD000399. 28056166 10.1002/14651858.CD000399.pub3 PMC6464941

[7] Neonatal Inhaled Nitric Oxide Study Group Inhaled nitric oxide in full-term and nearly full-term infants with hypoxic respiratory failure. N Engl J Med. 1997;336(9):597-604. doi:10.1056/NEJM199702273360901 9036320

[8] Clark RH, Kueser TJ, Walker MW, ; Clinical Inhaled Nitric Oxide Research Group Low-dose nitric oxide therapy for persistent pulmonary hypertension of the newborn. N Engl J Med. 2000;342(7):469-474. doi:10.1056/NEJM200002173420704 10675427

[9] Konduri GG, Solimano A, Sokol GM, ; Neonatal Inhaled Nitric Oxide Study Group A randomized trial of early versus standard inhaled nitric oxide therapy in term and near-term newborn infants with hypoxic respiratory failure. Pediatrics. 2004;113(3, pt 1):559-564. doi:10.1542/peds.113.3.559 14993550

[10] Pandit PB, Dunn MS, Colucci EA Surfactant therapy in neonates with respiratory deterioration due to pulmonary hemorrhage. Pediatrics. 1995;95(1):32-36. 7770305

[11] Willson DF, Thomas NJ, Markovitz BP, ; Pediatric Acute Lung Injury and Sepsis Investigators Effect of exogenous surfactant (calfactant) in pediatric acute lung injury: a randomized controlled trial. JAMA. 2005;293(4):470-476. doi:10.1001/jama.293.4.470 15671432

[12] O’Rourke PP, Crone RK, Vacanti JP, Extracorporeal membrane oxygenation and conventional medical therapy in neonates with persistent pulmonary hypertension of the newborn: a prospective randomized study. Pediatrics. 1989;84(6):957-963. 2685740

[13] Fletcher K, Chapman R, Keene S An overview of medical ECMO for neonates. Semin Perinatol. 2018;42(2):68-79. doi:10.1053/j.semperi.2017.12.002 29336834

[14] Kumar D, Super DM, Fajardo RA, Stork EE, Moore JJ, Saker FA Predicting outcome in neonatal hypoxic respiratory failure with the score for neonatal acute physiology (SNAP) and highest oxygen index (OI) in the first 24 hours of admission. J Perinatol. 2004;24(6):376-381. doi:10.1038/sj.jp.7211110 15116137

[15] Khemani RG, Rubin S, Belani S, Pulse oximetry vs. PaO2 metrics in mechanically ventilated children: Berlin definition of ARDS and mortality risk. Intensive Care Med. 2015;41(1):94-102. doi:10.1007/s00134-014-3486-2 25231293

[16] Khemani RG, Thomas NJ, Venkatachalam V, ; Pediatric Acute Lung Injury and Sepsis Network Investigators (PALISI) Comparison of SpO2 to PaO2 based markers of lung disease severity for children with acute lung injury. Crit Care Med. 2012;40(4):1309-1316. doi:10.1097/CCM.0b013e31823bc61b 22202709

[17] Rawat M, Chandrasekharan PK, Williams A, Oxygen saturation index and severity of hypoxic respiratory failure. Neonatology. 2015;107(3):161-166. doi:10.1159/000369774 25592054 PMC4405613

[18] Doreswamy SM, Chakkarapani AA, Murthy P Oxygen saturation index, a noninvasive tool for monitoring hypoxemic respiratory failure in newborns. Indian Pediatr. 2016;53(5):432-433. 27254060

[19] Howson CP, Kinney MV, Lawn JE, eds; March of Dimes, PMNCH, Save the Children, WHO Born Too Soon: The Global Action Report on Preterm Birth. Geneva, Switzerland: World Health Organization; 2012 https://www.who.int/pmnch/media/news/2012/201204_borntoosoon-report.pdf. Accessed September 10, 2018.

[20] Detry MA, Ma Y Analyzing repeated measurements using mixed models. JAMA. 2016;315(4):407-408. doi:10.1001/jama.2015.19394 26813213

[21] Di Fiore JM, Carlo WA Assessment of neonatal pulmonary function In: Martin RJ, Fanaroff AA, Walsh MC, eds. Fanaroff and Martin’s Neonatal-Perinatal Medicine. 10th ed Philadelphia, PA: Elsevier; 2015.

