# Supplementary material for: Evaluation of Oxygen Saturation Index Compared With Oxygenation Index in Neonates With Hypoxemic Respiratory Failure
Source: JAMA Netw Open. 2019 Mar 29;2(3):e191179. doi: 10.1001/jamanetworkopen.2019.1179 (PMC6450323; doi:10.1001/jamanetworkopen.2019.1179)
Supplement: Supplement. — eTable 1. Association of OI With OSI Using Multivariate Model eTable 2. Baseline Characteristics for Derivation and Validation Data Sets [file jamanetwopen-2-e191179-s001.pdf]

Supplementary Online Content

Muniraman HK, Song AY, Ramanathan R, et al. Evaluation of oxygen saturation index compared with oxygenation index in neonates with hypoxemic respiratory failure. *JAMA Netw Open*. 2019;2(3):e191179. doi:10.1001/jamanetworkopen.2019.1179

eTable 1. Association of OI With OSI Using Multivariate Model
eTable 2. Baseline Characteristics for Derivation and Validation Data Sets

This supplementary material has been provided by the authors to give readers additional information about their work.

© 2019 Muniraman et al. *JAMA Network Open.*

eTable 1: Association of OI With OSI Using Multivariate Model

	Estimates	95% CI	*P* value
OI	0.2868	0.2554, 0.3182	<.001
Pa_{CO_2}	−0.0032	−0.0291, 0.0228	.81
pH	0.9363	−2.9446, 4.8173	.64
Temperature	0.1747	−0.0070, 0.3564	.06

Abbreviations: OI: Oxygenation index, OSI: Oxygen saturation index

© 2019 Muniraman et al. *JAMA Network Open.*

eTable 2: Baseline Characteristics for Derivation and Validation Data Sets

	Derivation data (n=1018)	Validation data (n=436)	*P* value*
GA	28.93 ±5.07	29.01±5.23	.78
pH	7.29 ± 0.11	7.28 ±0.11	.13
P_{CO_2}	45.33 ±12.01	46.59 ±12.53	.07
P_{O_2}	59.34 ±32.17	61.18± 37.34	.34
Sp_{O_2}	93.29± 6.41	93.28±6.74	.97
Hb	14.23± 2.61	13.80 ±2.53	.004
F_{IO_2}	33.12 ±21.40	33.78±21.60	.59
OI	7.47± 8.98	7.30 ± 8.37	.74
OSI	4.10 ±4.54	4.09 ±4.35	.95

Abbreviations: F_{IO_2}, fraction of inspired oxygen; GA, gestational age; Hb: hemoglobin; OI, Oxygenation test; OSI, Oxygen saturation index; Sp_{O_2}, oxygen saturation measured by pulse oximetry.

**t* test.

© 2019 Muniraman et al. *JAMA Network Open.*
